# Sand Enteropathy—What We Know So Far and Why There Is Still So Much to Learn

**DOI:** 10.3390/ani16152324

**Published:** 2026-07-29

**Authors:** Lena Karlotta Lousberg, Ann Kristin Barton

**Affiliations:** 1 Equine Clinic Hochmoor, 48712 Gescher, Germany; 2 Veterinary Department, Freie Universitaet Berlin, 14163 Berlin, Germany

**Keywords:** horse, sand enteropathy, colic, geophagia, psyllium

## Abstract

Sand enteropathy describes pathophysiological conditions of the equine gastrointestinal tract due to the uptake and accumulation of sand. It is an increasingly recognized cause of gastrointestinal disease in arid geographic areas. Clinical signs can vary widely from colic, fever and inappetence to poor performance. Diagnosis based solely on clinical signs is nearly impossible. The current literature describes a variety of diagnostic aids, with radiographic imaging considered the gold standard. Therapeutic approaches aim at the evacuation of sand from the abdomen. This can be achieved by the administration of psyllium in connection with a laxative via nasogastric intubation. Several studies describe varying laxatives such as mineral oil or sodium-/magnesium sulphate. This narrative review includes 39 publications up to the year 2026. We aim to raise awareness among practicing veterinarians and highlight new research objectives to improve knowledge and treatment of gastrointestinal diseases related to sand accumulation.

## Simple Summary

Sand enteropathy is a term used to describe gastrointestinal disease associated with the accumulation of sand in the equine abdomen. The sand is mainly located in the large colon and, here especially, in the cranioventral part. It is ingested either voluntarily—called geophagia—or involuntarily with feed. The disorder is deemed an individual’s disease with no special preferences in terms of breed or sex. However, specific extensive breeds and management practices are described more often than others. As management practices tend to become more extensive, horses seem to have more access to sand. This review summarizes the current knowledge of this form of gastrointestinal disease, as well as its diagnostic and therapeutic options.

## 1. Introduction

The term sand enteropathy refers to the disruption of gastrointestinal function due to the ingestion and accumulation of sand. Sand is either ingested randomly or by targeted feeding on soil. 

Voluntary uptake of soil, geophagia, may be induced by boredom or deficiencies in mineral serum levels [1]. Licking sites visited frequently by horses were found to have high concentrations of iron and copper [1]. Both minerals were significantly lower in the serum of geophagic horses [2]. However, the authors of this previous study suggest that iron deficiency is unlikely to be the primary driver of geophagia. Instead, they discuss whether iron deficiency is observed secondary to copper deficiency, as this mineral is needed for the transport of iron through biomembranes, hence a decreased serum copper being the primary cause of geophagia. 

Involuntary sand intake can happen through feed and certain management practices [3,4,5,6,7,8]. Jurjanz et al. [9] found sward height after grazing to be a good indicator for the amount of ingested sand. Additionally, the quantity of daily herbage allowance was correlated negatively with the volume of sand ingested, ranging from 2.6% to 5.3% of daily roughage volume [9]. Moreover, the quality of soil can influence sand excretion. Sandy soil is associated with a higher sand excretion [6]. Other factors determining sand ingestion are either a combination of higher grass and sandy soil or bottom feeding and sandy soil [6]. Predisposed geographic areas include Scandinavia [4,5,10,11], Brandenburg (Germany) [12] and Switzerland [13] in Europe; California, Florida [4,14,15], the southwestern United States [15], Michigan [14], and British Columbia (Canada) [4] in North America; as well as western Australia, the Middle East, and Brazil [4].

Consumed sand primarily sediments and accumulates in the large colon [10,14,16] due to the slow transit of ingesta [14]. Sand can be found in each section of the large colon, with the ventral colon being described most frequently. There, it can cause mucosal damage, obstruction of the lumen as well as motility dysfunction [7,15,17].

Horses with sand enteropathy make up to 5% of referral colic cases worldwide [12,18,19]. However, a high regional variance is described [4,12,13,14,15] and prevalence can rise up to 11% in said geographic areas [20]. 

There has been a noted increase in clinical cases of sand enteropathy over the past 10 years [13], which has highlighted the need for accurate diagnosis and effective treatment.

## 2. Methods

To obtain as comprehensive an overview of the topic as possible, we searched the databases of PubMed Central^®^ (PMC) (https://pubmed.ncbi.nlm.nih.gov, accessed on 25 June 2006)—the free full-text archive of biomedical and life sciences journal literature at the US National Institutes of Health’s National Library of Medicine (NIH/NLM)—and the Thieme Vetcenter (https://vetcenter.thieme.de/home/startpage?lang=en, accessed on 25 June 2006) using the search terms ‘sand enteropathy’, ‘sand colic’, or ‘sand impaction’ linked to the term ‘horse’ to provide information on the main topic of this review. As research into sand enteropathy is very limited, no exclusion criteria were set, and so this narrative review includes all publications found on PubMed, including scientific articles, reviews, theses, and book chapters written in English or German up to June 2026.

References of the main relevant articles were searched for the complementary literature, covering topics of ‘geophagia’ and diagnostic approaches. These were subsequently added to the management software. Moreover, background information on the key components of therapy was conducted in the same manner, using keywords ‘adverse effects’, or ‘mechanism’ AND ‘sodium sulphate’, or ‘mineral oil’, or ‘psyllium’. Topic-specific reviews or case studies on laxatives were therefore included in this review’s literature as well.

The relevant literature was assembled, reviewed and managed using the software ‘Zotero’ (Zotero 9.0.4, © 2006–2018 Contributors). After the literature search, key information from each source was merged into a sorted document and incorporated into the manuscript. For better illustration of written information, original imaging produced during research work for the author’s own doctoral thesis (L.L.) was added. No generative AI was employed for either part of this work.

## 3. Clinical Appearance

Clinically, sand enteropathy affects individual horses rather than entire groups, with only isolated horses developing colic or other clinical signs [21]. There is no sex predisposition [4,15] and no single breed has an increased risk of sand accumulation. However, there are several breeds frequently mentioned in the literature: Quarter Horses [4,15,17], Shetland Ponies [4,13], Miniature Horses [4,17,20] and Finnhorses [4,5]. Graubner et al. suggested an association between sand enteropathy, a “good appetite” and management practices characterized by longer pasture or paddock turnout [13]. 

It is still unclear why some horses accumulate sand, while others completely excrete it in feces. Fintl et al. [22] found that the number of interstitial cells of Cajal, responsible for gastrointestinal motility patterns, is decreased in the colon of horses with obstructive disorders. It is assumed that mucosal inflammation and hypoxia lead to the damage of these cells and, as a result, to a decrease in gastrointestinal motility. This could factor in the larger sedimentation of ingested sand in these patients, but most certainly does not seem to be the only reason. 

Horses with sand enteropathy present with a wide range of clinical signs, the most common being acute, chronic or recurrent colic followed by diarrhea, weight loss, inappetence, poor performance, hyperesthesia and pyrexia [4,12,13,15,17]. Many horses show more than one clinical sign [5]. The combination of clinical signs may in some cases mimic equine gastric ulcer disease, thus being a valuable differential diagnosis [23]. The duration of symptoms range from one day to more than four weeks [17]. Horses with signs of colic or poor performance have larger accumulations than those showing other clinical signs.

On initial examination, patients had tachycardia [24] and decreased intestinal borborygmi [13,15,17]. Hematological examination showed a left shift in leucogram in 87% of examined horses, with either neutropenia or neutrophilia, as well as hyperlactatemia and some plasma electrolyte shifts [17].

## 4. Diagnosis

As clinical signs are nonspecific, a diagnosis cannot be based on the clinical examination alone. Additional examinations are required to confirm the presence of intestinal sand accumulation in suspected cases.

The following therapeutic options are described to aid the diagnosis of sand enteropathy (Table 1): 

### 4.1. Rectal Palpation

As most horses presenting with sand enteropathy show signs of colic, transrectal palpation often is a crucial part of the clinical workup. Sand can be palpated in feces or in the colon lumen [4,12,14]. 

There is a high possibility that sand may be out of reach during rectal examination as it mostly accumulates in the ventral abdomen, thus making diagnosis by transrectal examination more difficult. If sand itself is not palpated, there are rectal findings that could indicate the existence of a sand accumulation in the abdomen. In up to 54% of horses [13,25] a colon impaction was diagnosed by rectal examination. In some cases it might be challenging to differentiate between sand or ingesta filled impaction [14]. A meteorism of the large colon has been described in 51% of cases [25]. 

### 4.2. Fecal Glove Sedimentation Test

**Figure 1:**
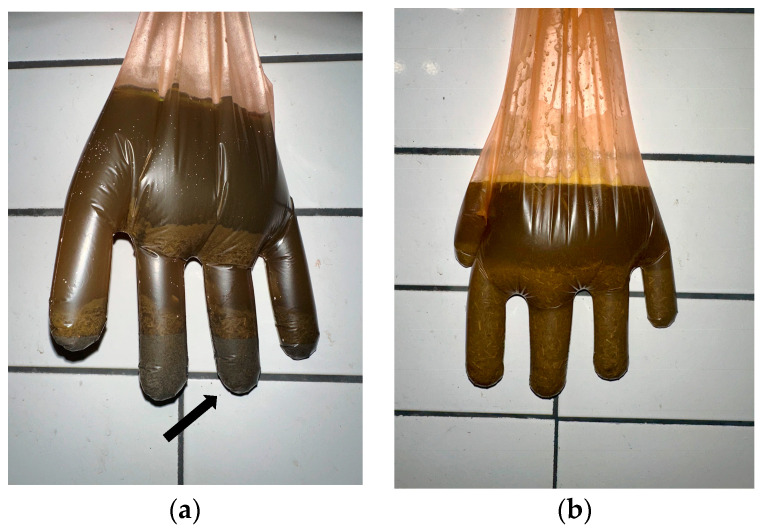
This image shows the fecal glove sedimentation test. Sand is clearly visible on the bottom of each finger, see arrow (**a**). A negative test is shown in image (**b**).

The principle of the fecal glove sedimentation test is to suspend a fecal sample in water, allowing sand particles to sediment (Figure 1). Husted et al. have standardized this test, using 200 g of feces and 1 L of water, letting the mixture rest for 20 min in a rectal examination glove before evaluation [6]. They score the amount of sand into five grades: 0: being no sand, 1: 0.5–5 mm of sand, 2: 5–10 mm of sand in at least one finger, 3: 10–20 mm of sand, and 4: over 20 mm of sand. This test was validated by comparing the results to the decantation of the same sample. A strong correlation between the results was found.

Hukkinen et al. examined the diagnostic utility of the fecal glove test for the diagnosis of sand enteropathy. They applied the standardized method of Husted et al. with a slightly modified scoring system. Rather than measuring the height of the sand sedimentation in each glove finger, each finger was assigned a score from 0 (no sand) to 3 (large amount of sand).The individual scores were added to obtain a total score ranging from 0 to 15. This test had a sensitivity of 83% and a specificity of 71% for diagnosing sand accumulations [26]. 

A later study showed that the sedimentation test was only positive in 34.3% of horses with sand accumulation [13], and another study found out that scores of the sedimentation test were similar between horses with different forms of colic [27]. Finally, the test was also positive in 64% of samples from non-colic horses [27].

The test is therefore useful for confirming the presence of sand accumulation, but it is not suitable for excluding it in horses with negative test results [4,26]. Its potential role in monitoring treatment response has also been discussed [3].

### 4.3. Abdominal Auscultation

Sand in the abdomen is described to make a distinct sound when auscultating the ventral abdomen caudal at the xiphoid process [3,12]. It makes a seashell-like sound when being moved by intestinal motility [3,4]. As large accumulations of sand are mostly seen with decreased intestinal borborygmi, the auscultation might not always be helpful in these cases. Additionally, the seashell-sound might as well occur in horses with small, clinically irrelevant accumulations, thus providing no information about the size or relevance of the sand accumulation [3].

### 4.4. Abdominal Ultrasonography

During ultrasound examination of the abdomen, sand can mostly be visualized in the ventral abdomen along the *linea alba* [12]. The colon wall looks hyperechoic with acoustic shadowing [3,16]. Korrolaien et al. evaluated the application of this method for the assessment and diagnosis of sand enteropathy [16]. On ultrasound the following aspects were evaluated: motility (scoring from 0: good to 3: bad), location (scoring from 0: no part against the ventral wall to 1: at least one part against ventral wall) and form and echogenicity of the ventral. More than half (59.4%) of the patients had decreased motility. In 56.3% of cases at least one part of the colon was in contact with the ventral abdominal wall. This correlates strongly with low motility findings. There were, however, differences in size and echogenicity. In this study abdominal ultrasound had an 87.5% sensitivity and 87.5% specificity for diagnosing sand enteropathy. More dorsal accumulations were not identifiable by ultrasound and appearance of the accumulation surface was not visible. Ultrasonography makes a helpful tool for determining sand in the ventral abdomen. However, its use for distinguishing between clinically relevant and clinically irrelevant sand accumulations cannot be recommended. 

Only one study has compared ultrasonographic and radiographic findings [16]. Further studies, particularly in field settings, are needed to evaluate clinical applicability.

### 4.5. Abdominal Radiography

Radiography of the abdomen is the gold standard in diagnosing sand enteropathy [4]. It can reliably identify accumulations that are out of reach of rectal palpation or negative in any of the other diagnostic aids [12,14,15].

On abdominal radiography, sand is depicted as a radiopaque mass, mostly located in the ventral abdomen [14,21]. Latero-lateral projections are acquired of the ventral abdomen [3,12,14,21]. They allow accurate assessment of the location, size and number of the accumulations [12], as well as the shape and the appearance of the dorsal surface [10,14,16].

**Figure 2:**
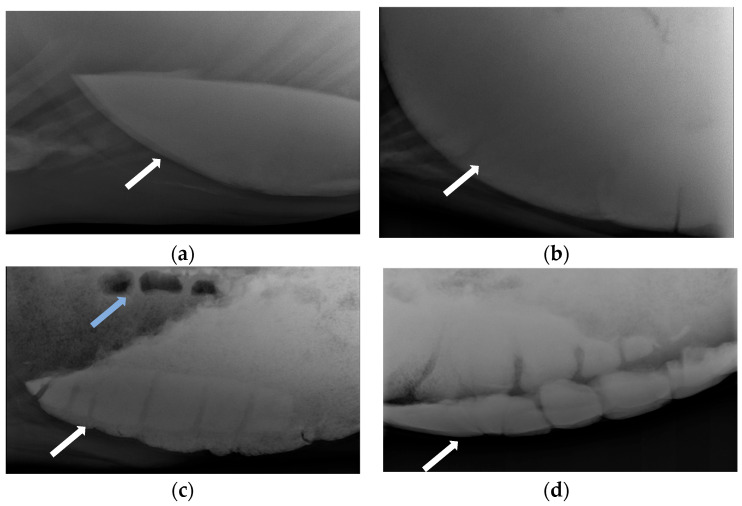
This figure shows different appearances of sand accumulation on abdominal radiography, see white arrow. In (**a**,**b**) the ventral margin is close to the ventral abdominal wall and no sacculations are visible. In (**b**) the dorsal border does not fit on the image. In (**c**,**d**) the sacculation of the ventral colon are clearly visible. (**c**) also shows some enclosed gas (blue arrow).

Several scoring systems have been developed to identify clinically relevant sand accumulations and to grade the severity of sand enteropathy [4,10,14,17,28]. Examples of differing radiographic appearence of sand accumulations are shown in Figure 2.

Niinstö et al. [5] classified the accumulations using their surface area in cm^2^. They determined accumulations larger than 75 cm^2^ to be clinically relevant and an accumulation smaller than 25 cm^2^ to be categorized as resolved. 

A different approach by Keppie et al. [14] established six categories to grade the accumulation. Categories were the number of accumulations, opacity compared to a rib, homogeneity of the accumulation, location of the accumulation, as well as height and width compared to the width of a rib. An overall score summing up the scores of each category was made. A maximum of 12 points could be reached. This study found a cut-off score of seven to have an 83% chance of a positive diagnosis of sand enteropathy. In addition, this score proved to have a good intra- and interobserver variance.

In the following years, Kendall et al. applied Keppie’s score to another study population. They found that the only parameters differing between colic and non-colic groups were length, width and homogeneity. Thus, they simplified the former score to a grading scheme based on the size of the largest visible accumulation. Grades range from 0, meaning no visible sand to 4: an accumulation larger than 5 × 15 cm^2^ or 15 × 5 cm^2^. Grades 1 and 2 were found in clinically healthy horses, whereas higher grades were associated with clinical signs of colic. Another reason for simplifying the Keppie et al. score was the difficulty in identifying and measuring the ventral part of the rib on the radiographs.

One study used all three scores and found the results to be correlating [13]. Though, observers preferred Keppie’s score, since it had the most categories to differentiate the sand accumulations.

Another study employed the percentage of luminal obstruction of the colon to grade the sand accumulation [17]. A score of 0 indicated no visible sand, whereas scores of 1, 2, and 3 corresponded to <30%, 30–60% and >60% obstruction of the colonic lumen on radiographs, respectively.

Another recent study related the surface area of the accumulation to the patient’s weight [29]. A cut-off value of 0.21 cm^2^/kg to cause clinical signs was found. While this study is the first to match the amount of sand to bodyweight, the findings should be applied with care, as miniature horses were overrepresented.

Three studies describing the use of radiography for the diagnosis of sand enteropathy were retrospective [14,21,29]. Consequently, radiographic imaging was not standardized across patients. However, one of these studies compared the radiographic findings with intraoperative findings, thereby validating the grading system developed in that study [14].

## 5. Therapy

For sand enteropathy, the overall therapeutic aim is the evacuation of sand from the abdomen [4]. This can be achieved medically or surgically.

The first step should be the stabilization of the patient according to the findings on initial exam. This includes administration of analgesics [3,4,17], compensation of hematological deviations [3,4], intestinal rehydration, as well as support of gastrointestinal motility [4,17]. General prognosis is 90–95% of long-term survival [4,13,15]; however, there are minor differences between the treatment options.

### 5.1. Medical Therapy

Medical treatment consists of fluid therapy, administered enterally or intravenously combined with a laxative [3,4,12,15,17,29]. The vast majority (72.5%) of cases can be treated successfully with conservative management [15]. Key components of laxative treatment are magnesium sulphate [4,12,13,15], mineral oil [12,13,15,30] and psyllium [4,17,31]. 

Psyllium is regarded the key stone in the therapy of sand enteropathy. In contact with water it develops a hydrocolloid gel, which subsequently collects sand and promotes excretion by increasing fecal volume [32]. It is administered at a dose of 1 g/kg BW [11,12,13,30] either via nasogastric intubation or in feed [4,11,30]. While one study questions the efficacy of psyllium in removing sand [31], it has been established as a standard component of therapy in many successful studies [11,13,15,17,30,33,34]. However, it is not recommended for long-term-therapy, as the intestinal microbiome adapts by increasing psyllium-degrading bacteria [32]. Application can be performed through feed or via nasogastric intubation. The latter is considered more effective and should be preferred in hospitalized horses [11].

Magnesium sulphate is an osmotic laxative, which increases the osmotic pressure inside the intestinal lumen, causing water to move into the lumen down the osmotic gradient [12]. For the treatment of sand enteropathy it is used at a dose of 1 g/kg BW [33]. In the European literature the use of sodium sulphate is described as well [13]. 

The application of a combination of magnesium sulphate (1 g/kg BW) and psyllium (1 g/kg BW) proved to be more effective than either component alone [33]. Most horses of the combination group evacuated the sand in 4 days (75%), compared to only 25% of the others. These findings were subsequently verified in a second study [34]. These studies were both prospective and standardized studies, with the same inclusion criteria. Thus, they are comparable and reliable in supporting the efficiency of a combination of psyllium and magnesium sulfate for sand clearance. Another frequently used laxative is mineral oil [12,13,15,17,30]. It aids by lubricating the impaction [12]. The combination of psyllium (500 g b.i.d.) with mineral oil (2 L s.i.d.) was found to be more effective in the clearance of sand than mineral oil on its own [30]. Sand was quantified by measuring crude ash in feces, and horses were screened for pre-existing sand accumulations using ultrasonography only. Therefore, the results of this study may not reflect complete clearance of gastrointestinal sand accumulations.

In an Australian study, a combination of psyllium (1 g/kg BW), magnesium sulphate (1 g/kg BW) and mineral oil (6–8 mL kg BW) was successful in 81% of the horses and cleared more than 85% of the sand in 90% of the animals. [28].

Two other products have been tested in the evacuation of sand. Alonso et al. [35] compared the success of the administration of carboxymethylcelluose (1 g/kg BW) to psyllium or water via nasogastric intubation. Most excretion of sand was reached with the application of 8 L of water.

Another supplement containing pre- and probiotics and psyllium showed an increased sand output after 3 days of feeding [36].

Complications described with medical therapy are recurrent colic (at least one more episode of colic in 51% of cases [15]), diarrhea, colitis [13], fever or thrombophlebitis [15]. Especially, therapy with magnesium sulphate is correlated with diarrhea [15]. Furthermore, there is one case report about a gastric rupture after feeding a supplement containing psyllium at 4 times the recommended dose [37].

Nevertheless, success rates of medical therapy described in the literature is 86–95.7% [3,12,15,17].

### 5.2. Surgical Therapy

Surgical therapy is needed in 14–31% of cases [3,13,15,33]. Factors requiring surgical therapy are meteorism of the colon, suspicion of displacement, persistent impaction and uncontrollable pain levels [3,15,17,25,33]. Surgical approach should be preferred especially in horses with uncontrollable pain [4].

For the surgical approach, a median laparotomy is performed. The primary finding during surgery includes the impaction of the colon [15]. Nevertheless, an additional lesion of the gastrointestinal tract, such as right dorsal displacement, and large colon volvulus 180° and 360°, was described in almost half (47.4%) of the cases [15]. Other findings included impactions with a phytobezoar, intestinal adhesions, volvulus of the small intestine as well as full-thickness ruptures of the large colon [25]. 

For the evacuation of sand, the colon is exteriorized and an enterotomy of the pelvic flexure is executed. Care must be taken in chronic cases of sand enteropathy, as the damaged mucosal wall is prone to ruptures during movement of the sand-filled colon [3,12].

The short-term prognosis is described to be 95% of treated cases [15,25]. The rates for long-term survival are described to be a little lower, with 87.5% [15]. Moreover, 16.7% of surgically treated horses experienced at least one more colic episode after discharge [25].

### 5.3. Prevention/Prophylaxis

Although listed under therapy, prevention is an essential component of long-term management. To prevent new episodes of sand enteropathy, changes in management of affected horses are crucial.

As already described, there are certain risk factors for sand-uptake like boredom and mineral deficits [1], lower daily herbage allowance [9], sandy soil and bottom feeding [6]. To properly treat this disease all those points mentioned above should be considered for management optimization. Several preventive strategies have been proposed in the literature, including limiting pasture time, especially on empty pastures, or adding roughage supply to the pasture [6]; preventing bottom feeding, or at least the contact of feed with the soil, using hay racks or mats [12,31]; supplementing minerals [1,2]; and separating horses with lower positions in the herd during feeding times [5]. Preventing further sand ingestion is likely essential for complete recovery and reducing the risk of recurrence (Table 2).

## 6. Discussion and Conclusions

Sand enteropathy is a complex disease that clinicians are likely to encounter with increasing frequency in the future. However, diagnosis can be challenging, as clinical signs are often subtle. Moreover, sand enteropathy should remain an important differential diagnosis in horses presenting with colic, particularly when obstipation and/ or meteorism are present. 

To date, explanations of why specific horses are prone to the accumulation of sand while others seem to be able to fully excrete it are not available. To completely understand this disease, further investigation into its pathophysiology is essential. Fintl’s research [22] on interstitial cells of Cajal is a crucial advancement. However, it cannot answer the question of cause and effect, and thus, a tool to predict which horses are at higher risk is still missing. If an association between intestinal hypomotility due to a reduced number of pacemaker cells and sand accumulation were confirmed, pharmacological stimulation of intestinal motility could be considered as a short-term therapeutic strategy, alongside long-term management aimed at restricting access to sand. Another interesting point to consider could be the influence of diet on the risk of sand accumulation. A recently published prospective study found a statistical difference between the motility of the sternal flexure of horses fed hay or pelleted food [38]. In this study horses that were fed pelleted food had a higher motility than those fed only hay. One factor that aligns with the thought of dietary influence could be the influence of the intestinal microbiome. The motility may also be altered by the weight of the sand accumulation itself or by inflammatory processes that predispose the horse to sand accumulation initially or arise from the irritation of sand particles on the mucosa. Last but not least, the characteristics of the accumulated sand may play an important role, including grain size and surface structure. Since horses kept on sandy soil are more likely to develop sand enteropathy, looking at soil characteristics could be an interesting approach to understand reasons for accumulation. The mineral composition of these accumulations could also factor in the likelihood of sedimentation and thus on therapy outcome. It would be therefore interesting to compare sedimented sand between geographic origins, as soil is composed differently in different regions on this planet. Unfortunately, the evidence supporting these concepts is currently lacking; therefore, further research, in particular prospective studies, is needed.

Radiography of the abdomen is still the most reliable method to not only diagnose, but also assess the severity of the sand enteropathy [4]. There are a few radiographic scoring systems developed to differentiate between clinically relevant and irrelevant accumulations [14,21,29]. Existing systems seem to lack detailed grading of large accumulations. The maximum score should reflect on the largest possible accumulation, so that large accumulations can additionally be divided into subgroups. Next steps in research should be the development of a scoring system or an addition to an existing system that not only differentiates by clinical relevance but also determines therapy length and prognosis. 

Unfortunately, the use of radiography is largely limited to referral hospitals, as high exposure settings are required to detect sand in the equine abdomen. These settings are difficult to achieve with ambulatory radiography equipment. Other diagnostic tests are lacking in specificity and sensitivity and should not be used to classify accumulations for severity of sand accumulations. Furthermore, therapy success can only be fully proven by abdominal radiography. Therefore, horses with sand enteropathy are likely to be underdiagnosed in the field. More research is mandatory to find a reliable diagnostic system applicable for ambulatory practice to identify and assess sand enteropathy.

The number of horses presenting with sand enteropathy appears to be increasing, while risk factors such as drought are unlikely to diminish in the future. Therefore, further research is essential. 

Therapeutic recommendations in the literature are becoming more consistent [4,12,24]. So far, the combination of psyllium and a laxative seems to be the most effective therapy option [30,33]. There are different laxatives in use, which have not yet been tested against each other. Unfortunately, efficacy measuring methods applied in the different studies are not comparable, and furthermore, existing laxatives have known side-effects such as diarrhea, colic-like symptoms and malabsorption [39]. This makes the choice of the right treatment regime for treating veterinarians very difficult. 

Finally, Entwistle et al. [28] mention a large number of non-responders, especially in miniature breeds, and these miniature breeds are frequently mentioned in the population of horses presented with sand enteropathy. So, the improvement of therapy in these high-risk breeds is needed. An increase in administration frequency of psyllium and the laxative to twice daily or a switch towards new laxatives could be an interesting approach.

To conclude, in the upcoming years, research should focus on improving the understanding of the pathophysiology of sand enteropathy, developing diagnostic aids applicable for mobile veterinary service, and finding new treatment options suitable for miniature breeds and free of adverse effects. At this point, the evidence base comparing the efficacy and adverse effects of different treatment regimens is weak, so future research should also focus on determining the most effective treatment option by comparing established methods in prospective studies and different populations of horses, as important differences may exist between breeds, feeding practice, sand quality in different regions and other confounding factors.

## Figures and Tables

**Table 1:** Diagnostic options for sand enteropathy—overview.

Diagnostic Tool	Sensitivity & Specificity	Application	Limitation
Rectal palpation	51–54% [13,25] (sensitivity)	Palpation of either sand or subsequent gastrointestinal pathologies	Sand accumulation may be out of reach
Fecal glove sedimentation test	83% sensitivity 71% specificity [26]	Solution of 200 g of feces in 1 L of water, rest for 20 min	Useful for positive diagnosis, but not for exclusion
Auscultation	No data	Seashell-like sound behind xiphoid process	Larger accumulations show lower intestinal borborygmi, sound does not reflect size of sand accumulation
Abdominal sonography	87.5% sensitivity 87.5% specificity [16]	Ventral abdomen, along linea alba	No information about size, higher accumulation cannot be visualized
Abdominal radiography	Gold standard	Ventral abdomen	Not applicable with ambulatory X-ray equipment

**Table 2:** Overview on current knowledge on sand enteropathy, focusing on risk factors, clinical appearance, diagnostic approach, treatment and prevention.

Risk factors	Geophagia: Boredom or mineral deficits
	Management practices: Empty pastures, low daily herbage allowance, sandy soil, bottom feeding, high grass
Clinical appearance	Colic, diarrhea, weight loss, inappetence, poor performance, hyperaesthesia, pyrexia
Diagnostic approach	Rectal palpation Fecal glove sedimentation test Auscultation Abdominal sonography Abdominal radiography
Treatment options	Medical therapy: Psyllium (1 g/kg BW) per feed or nasogastric intubation psyllium (1 g/kg BW) + magnesium sulphate (1 g/kg BW) psyllium (500 g) + mineral oil (2 L) psyllium (1 g/kg BW) + mineral oil (6–8 mL/kg BW) + magnesium sulphate (1 g/kg BW)
	Surgical therapy: Pelvic flexure enterotomy
Prevention	Improvement of management practices, limiting access to sand Ensuring sufficient herbage supply Exclusion of mineral deficits

## Data Availability

No new data were created or analyzed in this study. Data sharing is not applicable to this article.
